# CARDIOVASCULAR DISEASE RISK AND FALLS IN MEN AND WOMEN LIVING WITH AND WITHOUT HIV: MACS/WIHS COMBINED COHORT STUDY

**DOI:** 10.1093/geroni/igae098.3817

**Published:** 2024-12-31

**Authors:** Elizabeth Vasquez, Mark Kuniholm, Allison Appleton, Anjali Sharma, Michael Yin, Todd Brown, Phyllis Tien, Deborah Gustafson

**Affiliations:** University at Albany, SUNY, Rensselaer, New York, United States; University at Albany (SUNY), Rensselaer, New York, United States; University at Albany, Albany, New York, United States; Albert Einstein College of Medicine, Bronx, New York, United States; Columbia University, New York, New York, United States; Johns Hopkins, Baltimore, Maryland, United States; UCSF, San Francisco, California, United States; State University of New York Downstate Health Sciences University, BROOKLYN, New York, United States

## Abstract

Falls risk assessment tools underestimate the risk among People Living with HIV (PLWH). The aim of this cross-sectional analysis was to measure associations between American College of Cardiology/American Heart Association (ACC/AHA) 10-year cardiovascular disease (CVD) risk scores and history of falls in a multi-center United States cohort of PLWH and similar people living without HIV (PLWOH). CVD risk was categorized as low, borderline, intermediate and high (< 5%, 5-7.4%, 7.5-19.9% and ≥20%, respectively) and history of falls was ascertained using a standardized protocol across study sites. Among n=2728 participants (Men, n=939, 578 MLWH/361 MLWOH; (Women, n= 1789, 1274 WLWH/515 WLWOH), median ages were 58 (IQR:51-65) and 55 (IQR:49-60) years for men and women, respectively. Median CVD risk within 10 years was 11% (IQR:6%-18%) and 5% (2%-11%) for men and women, respectively. All analyses were adjusted for age and stratified by sex and HIV status. Higher CVD risk was associated with increased history of falls (high vs low CVD risk: OR=2.31; 95%CI:1.16,4.62; intermediate vs low CVD risk OR=2.13; 95%CI:1.11,4.07) among MLWOH. Findings were similar in MLWH after adjustment for CD4+ T cell count and HIV-1 RNA detectability (<=200). In WLWH, only high CVD risk was associated with falls (OR:1.76; 95%CI:1.13,2.7), while among WLWOH, no association of CVD risk with falls was observed when compared with a low-risk score (< 5%). Results suggest CVD risk scores may be beneficial to guide providers’ lifestyle or clinical management recommendations to decrease risk of falls and preserve and/or improve quality of life among MLWOH, MLWH, and WLWH.

